# The effect of silver nanoparticles, zinc oxide nanoparticles, and titanium dioxide nanoparticles on the push-out bond strength of fiber posts 

**DOI:** 10.4317/jced.56126

**Published:** 2020-03-01

**Authors:** Zahra Jowkar, Yekta Omidi, Fereshteh Shafiei

**Affiliations:** 1 Assistant professor, Oral and Dental Disease Research Center, Department of Operative Dentistry, School of Dentistry, Shiraz University of Medical Sciences, Shiraz, Iran; 2 Undergraduate Student, Department of Operative Dentistry, School of Dentistry, Shiraz University of Medical Sciences, Shiraz, Iran; 3 Professor, Oral and Dental Disease Research Center, Department of Operative Dentistry, School of Dentistry, Shiraz University of Medical Sciences, Shiraz, Iran

## Abstract

**Background:**

This study was undertaken to investigate the effect of intraradicular dentin pretreatment with silver nanoparticles (SNPs), zinc oxide nanoparticles (ZNPs), and titanium oxide nanoparticles (TNPs) on the push-out bond strength (PBS) of fiber posts to root dentin using two types of resin cements.

**Material and Methods:**

Eighty single-rooted human premolar roots were randomly divided into eight groups after endodontic treatment and post space preparation, according to the type of intraradicular dentin pretreatment with different nanoparticle solutions (n=20). The groups included no pretreatment (control) and pretreatments with SNPs, ZNPs, and TNPs. Each group was divided into 2 subgroups (n=10) according to cementation: Excite DSC/Variolink II and ED Primer II/Panavia F2.0. The PBS values were measured in different areas of the post space. The data were subjected to the three-way analysis of variance and Tukey tests (p=0.05).

**Results:**

The SNP-pretreated groups showed significantly higher PBS values than the other groups. No significant differences in PBS were noted among the control, ZNP-pretreated, and TNP-pretreated groups. There was no significant difference in the PBS of the fiber posts with respect to cement type. The PBS was significantly lower in the apical region than in the other two regions. There was no statistically significant difference between the PBSs of the cervical and middle thirds.

**Conclusions:**

Intraradicular dentin pretreatment with SNPs, TNPs, and ZNPs did not interfere with the PBS of the fiber posts. The best result was obtained for the SNP-pretreated groups for both types of cement. Also, the lowest PBS was found in the apical third of the root canal spaces.

** Key words:**Fiber post, nanoparticles, push-out bond strength.

## Introduction

Fiber-reinforced composite posts (also known as ‘fiber posts’) are frequently used in endodontically treated teeth with little coronal structure. Fiber posts have many favorable properties including aesthetic advantages, biocompatibility, an elastic modulus which is close to dentin, compatibility with Bis-GMA-based resin, resistance to corrosion, and high flexural strength (1,2). However, debonding is still the most common cause of failure associated with fiber posts (1). A highly durable bond between resin cement and root dentin is essential for post retention and survival/success of the restoration. Degradation of the exposed collagen fibrils along the base of the hybrid layer by matrix metalloproteinases (MMPs) and cysteine cathepsins is one of the most important mechanisms known to be responsible for bond degradation over time (3). The role of bacterial acids in the process of MMP activation has been indicated previously (4). Bacteria may still persist in the root canal system even after careful chemo-mechanical preparation and obturation of the root canals (5). One proposed method for reducing intracanal bacteria is to use antibacterial irrigants before fiber post cementation (6). However, the antibacterial irrigant should not negatively affect the bond strength of fiber posts to root dentin (6).

Effective dentin hybridization which is necessary for the bonding of fiber post to radicular dentin is affected by root regions, irrigants, adhesive systems, and post space preparations (6). One of the most common root canal irrigating solutions is sodium hypochlorite (NaOCl) which has antimicrobial properties and the ability of organic tissue-dissolving. However, it may adversely affect the adhesion of luting agents because it may cause collagen degradation and inhibit the polymerization of the resin materials (7,8). Chlorhexidine is another irrigating solution. Besides antimicrobial properties, CHX has substantivity and the ability of affecting the longevity of the bonding interface and inhibiting dentin matrix metalloproteinases (MMP) (9). However, a disadvantage reported for CHX is its probable leaching out of the hybrid layers within 18 to 24 months (10). It could also have some negative effects on the interface permeability and bond strength of fiber post to root dentin (6).

Recently, metal nanoparticles have gained popularity in various dentistry fields. They have unique properties and structures such as small size, large proportion of surface atoms, high surface energy, and very high surface to volume ratio. In addition, they have broad-spectrum bactericidal and virucidal properties with less chance of bacterial resistance compared to the majority of commercially available antibiotics (11,12). Silver nanoparticles (SNPs) possess confirmed biocompatibility especially in lower concentrations and long-term and broad-spectrum antibacterial and antiviral properties even in low concentrations (13,14). Moreover, SNPs have 25-fold higher antibacterial efficacy than chlorhexidine (CHX) (15). Dentin pretreatment with SNP positively affected the bond strength of etch-and-rinse and self-etch adhesives in a previous study (16). Also, no negative effect on the interface permeability and bond strength values of fiber posts to intraradicular dentin was observed when silver nanoparticle solution was applied as an irrigation agent in post space prior to fiber post cementation process (6).

Zinc oxide nanoparticles (ZNPs) demonstrated potent antibacterial effects against several types of dental plaque bacteria including *S. mutans* and *Lactobacillus* (17). Also, titanium dioxide nanoparticles (TNPs) have recently been used in dentistry because of their bactericidal effect (which is even better than that of chlorhexidine), pleasing color, and high biocompatibility (15,18).

It was shown in a previous study that dentin and enamel pretreatments with SNPs, TNPs, and ZNPs did not have any adverse effect on the bond strength values of composite resin to coronal dentin and pretreatments with SNPs showed the best results (19).

However, the effect of post space pretreatment with different nanoparticle solutions on the push-out bond strength (PBS) of fiber post to root dentin has not been evaluated. Thus, the aim of the current study is to evaluate the effect of applying silver, zinc oxide, and titanium dioxide nanoparticles on the PBS of fiber posts to root canal dentin.

## Material and Methods

**Figure 1:**
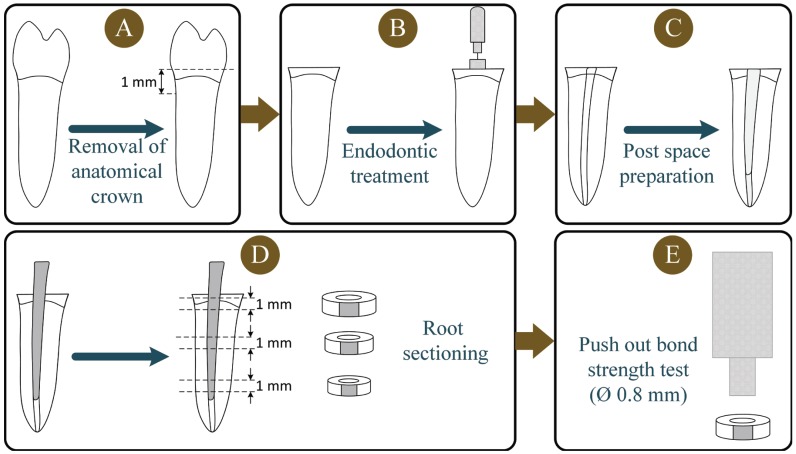
Schematic representation of the specimen preparation procedures: (A) Decoronation of the tooth at 1mm above cemento-enamel junction, (B) Endodontic treatment, (C) Post space preparation, (D) Sectioning the root to obtain a 1-mm-thick slice from each root region (apical, middle, and coronal), (E) Push-out bond strength test.

Ninety caries-free single-rooted human lower premolars (with mature apices and without root curvature) which were extracted for orthodontic or periodontal reasons were utilized in this study. The research protocol was approved by the Research and Ethics Committee of Shiraz University of Medical Sciences, (Protocol #97-01-03-18386). All of the procedures of this experimental study were done by a blinded calibrated operator. After cleaning the teeth with a periodontal curette, they were stored in a 0.5% chloramine solution at 4° C for no longer than one month before use. A water-cooled low-speed cutting machine (Mecatome T201 A, Presi, Grenoble, France) was used to remove the anatomic crowns of all teeth 1 mm above the cementum-enamel junction (Fig. 1). Endodontic treatment was performed with a step-back preparation technique using stainless steel K-files and #2 to #4 Gates-Glidden burs (Moyco Union Broach, York, PA, USA) under copious irrigation with a 1% sodium hypochlorite solution throughout the instrumentation. The lateral condensation technique was performed using gutta-percha cones (GC, Tokyo, Japan) and AH Plus resin sealer (Dentsply, York, PA, USA) for obturation of the prepared root canals. The obturated roots were then stored in distilled water at 37ºC in a 100% relative humidity for 48 hours. After storage, post spaces of approximately 11 mm in length were created with No. 2 drills from the respective post manufacturer by the same operator using a low-speed handpiece. The apical 5 mm of the gutta-percha filling remained intact in each specimen which was confirmed by radiography. The roots were randomly divided into eight experimental groups (n=10). The fiber posts (FRC Postec Plus No. 2, Ivoclar Vivadent, Schaan, Liechtenstein) were tried in the prepared depth to verify a passive fit in it.

The specimens were divided into eight groups according to the resin cements and intraradicular dentin pretreatment with SNPs, ZNPs, and TNPs (purchased from ASEPE Company, Tabriz, Iran) with particle sizes of 20 nm, <50 nm, and 20 nm, respectively (n=10). The fiber posts were cemented with an etch-and-rinse cement (E&R; Excite DSC/Variolink II, Ivoclar-Vivadent, Schaan, Liechtenstein) in four experimental groups. A self-etch cement (SE; ED Primer II/Panavia F2.0, Kuraray, Osaka, Japan) was used for post cementation in the other four groups. For each cement, one group was considered as the control group in which the cement was applied according to the manufacturer’s instructions without intraradicular dentin pretreatment. In groups E&R +SNPs, E&R+ZNPs, and E&R+TNPs, surface pretreatment was done with SNPs, ZNPs, and TNPs, respectively, for one minute before acid etching (19). Then the prepared post space was rinsed with distilled water thoroughly for one minute. In groups SE+SNPs, SE+ZNPs, and SE+TNPs, surface pretreatment was done with SNPs, ZNPs, and TNPs, respectively, for one minute before acid etching. Then the prepared post space was rinsed with distilled water thoroughly for one minute.

The post space pretreatment with three nanoparticle solutions (SNPs, ZNPs, and TNPs) was used in the SE groups (SE+SNPs, SE+ZNPs, and SE+TNPs groups) prior to the application of ED primer II. After cleaning the post spaces, they were gently air-dried with an air syringe and paper points. In each experimental group, the cement was applied on the post and into the canal. Then, the post was seated into the canal with a slight vibratory motion and firmly pressed for 5 to 10 s and the excess cement was removed. Light polymerization was performed with a light-curing unit (VIP Junior, Bisco, Schaumburg, IL, USA) at 600 mW/cm2 through the cervical portion of the root for 40 seconds at the buccal and lingual surfaces totaling 80 seconds of light exposure. Finally, a resin-modified glass ionomer cement (GC Fuji II LC, GC Corporation Tokyo, Japan) was used to create a tight coronal seal. The specimens were stored in distilled water at 37°C for 24 hours.

-Push-out Test and Failure Mode Analysis

After the storage period, the bonded roots were embedded in acrylic resin and sectioned perpendicular to their long axis using a slow-speed cutting machine (Mecatome T201 A, Presi, Grenoble, France) to obtain a 1-mm-thick slice from each root region (apical, middle, and coronal). Therefore, the sample size was 30 for each group (10 for each root region in each group). The first coronal slice was discarded because of the presence of excess cement. A digital caliper (Digimess Direct, Sao Paulo, Brazil) was used to verify the thickness of each root section. The push-out test was performed immediately for each obtained slab using a universal testing machine (Instron Z020, Zwick Roell, Ulm, Germany). The compressive load was applied at a crosshead speed of 0.5 mm/min on the center of the post in an apico-coronal direction until the shear stresses along the bonded interface dislodged the post. The post segment was loaded with three punch tip diameters (1 mm, 0.8 mm, and 0.7 mm) depending on the diameter of the post in each root region slice centered on the post segment without any contact with the surrounding dentin surface. The PBS for each post segment was calculated by dividing the load at failure in Newton by the interfacial area (A) of the post fragment which corresponded to the bonded area in square millimeters (mm2). The bonded area was calculated as the lateral surface of a truncated cone using the following formula: A = π (R + r) [h2 + (R - r)2] 0.5, where π is 3.14, R and r represent coronal and apical post radii, respectively, and h is the section height.

The failure mode was analyzed under a stereomicroscope (Carl Zeiss, Oberkochen, Germany) at 40× magnification for each debonded specimen and categorized as follows: (A) Cohesive failure in dentin, (B) Cohesive failure in the cement or post, (C) Adhesive failure between the cement and the dentin, (D) Adhesive failure between the cement and the post, (E) Mixed failures consisting of a combination of two or more failure modes.

The normality assumption was assessed using the Kolmogorov-Smirnov test. The normality assumption was held in all groups. The data were statistically analyzed using a three-way analysis of variance (ANOVA) model to evaluate the effects of three main factors (cement type, irrigant or intraradicular dentin pretreatment, and root region). Tukey’s post-hoc test was used to compare the PBS values of different groups. All the analyses were performed using SPSS software version 17 (SPSS Inc, Chicago, USA). P-values less than 0.05 were considered statistically significant.

## Results

The mean PBS values and standard deviations (SD) in MPa are shown in Table 1. The three-way ANOVA indicated that none of the two-way and three-way interaction effects were significant (all *p* values > 0.05) (Table 2). Therefore, the main effect analysis was performed to compare the mean PBS values between different groups. The comparisons using the post hoc Tukey test are shown in Table 3.

**Table 1:**
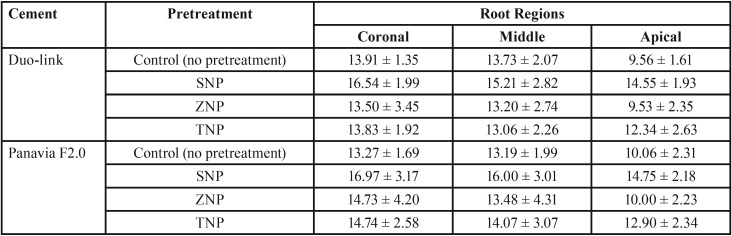
Push-out bond strength (mean ± standard deviation) (MPa) in designated thirds of intraradicular dentin pretreated with different irrigation solutions (n=10).

**Table 2:**
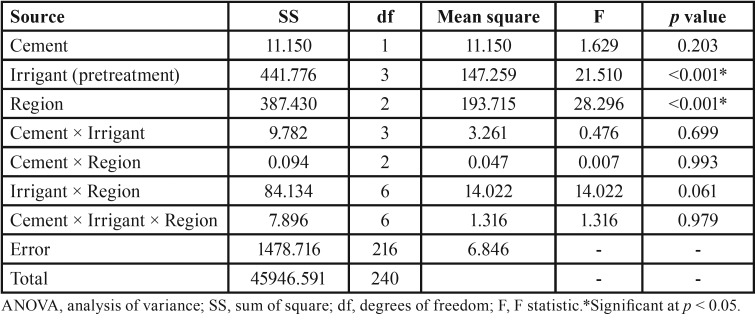
Results of the three-way AVOVA.

**Table 3:**
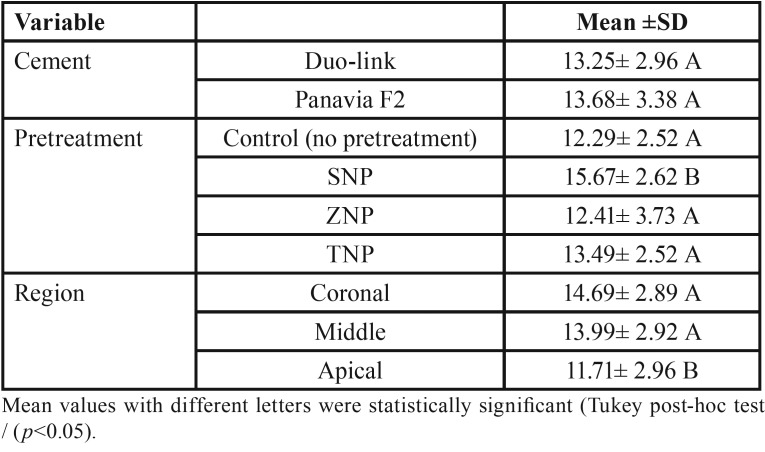
Estimated marginal means of PBS values (±Standard Deviations) for all cements, intraradicular dentin pretreatments, and root regions.

The PBS of SNP-pretreated groups was significantly higher than that of the corresponding control, ZNP-pretreated, and TNP-pretreated groups (*p* values<0.001). No significant differences in PBS were noted among the control, ZNP-pretreated, and TNP-pretreated groups (*p* values >0.05).

There was no significant difference in the PBS of fiber posts with respect to cement type (etch-and-rinse vs. self-etch) (*p*>0.05).

The PBS in the apical region was significantly lower than that of the other two regions (coronal and middle) (*p* <0.05). There was no statistically significant difference between the PBSs of the cervical and middle thirds (*p*>0.05).

The results of the failure modes of the study groups are displayed in Table 4. The predominant failure mode was the mixed failure in all the experimental groups.

**Table 4:**
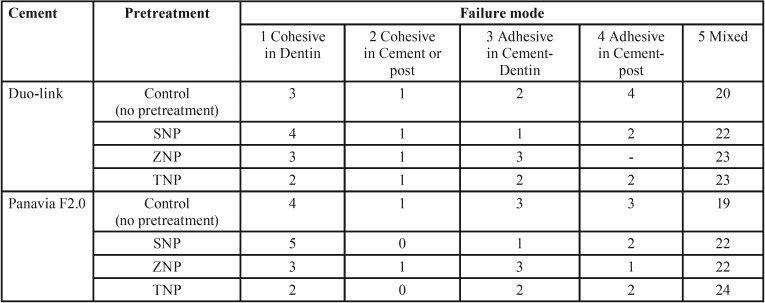
The failure mode of specimens of the study groups after the PBS test for each experimental group.

## Discussion

This study was conducted to evaluate the PBS of the fiber post to root canal spaces in different thirds of the intraradicular dentin after pretreatment with SNP, ZNP, and TNP solutions. According to the results of the present study, the intraradicular dentin pretreatment with SNP resulted in significantly higher PBS values compared to the control, ZNP-pretreated, and SNP-pretreated groups. No negative effect was observed following intraradicular dentin pretreatment with ZNP and TNP. Also, the root third affected the PBS values. The lowest mean bond strength value was observed in the apical third. Resin cement type did not affect bond strengths significantly.

Various antibacterial irrigation solutions have been used before fiber post cementation mainly to clean and disinfect the root canal system and/or to condition the dentin. Because of the observed decrease in the bond strength of fiber post to root dentin following the application of common irrigation solutions such as chlorhexidine and NaOCl, several attempts have been made to find alternative irrigation solutions with antibacterial properties (6,8). Therefore, different nanoparticle solutions were used as intraradicular dentin pretreatments before post cementation in the current study. None of the nanoparticle solutions interfered with PBS in the present study.

Two typical tests to assess the retention of posts in the root canal space are microtensile pull-out and push-out tests. The push-out test is based on measuring the shear stress (which is comparable to the stresses under clinical conditions) in the post-cement interface and in the dentin-cement interface (20). The micro-push-out bond strength test demonstrated more homogenous stress distribution, less variability in mechanical testing results, lower standard deviation, and fewer pretest failures compared to the microtensile bond strength test (21). In the current study, the push-out test was carried out 24 hours after fiber post cementation to allow complete polymerization of the luting material during this period.

Two types of resin cements (E&R and SE) were used in this study. No significant difference was observed between the two resin cements used in the present study. A previous study showed that the self-adhesive (SA) cement had higher immediate bond strength than the E&R and SE resin cements. No significant difference was found between the PBS values of the E&R and SE resin cements which was in line with the present study. However, after aging, the bond strength of the three types of resin cements decreased with no significant differences among the resin cements (22). The application of the E&R and SE resin cements is accompanied by some difficulties in the root canal. E&R resin cement adhesion processes are accompanied by some difficulties such as homogeneous etching, complete rinsing of the etchant, the optimal degree of dentin wetness needed, thorough resin penetration, the homogeneous application of the adhesive/primer, and the complete evaporation of the solvent from the primer/adhesive applied in root canal spaces (22). SE resin cements may also encounter the last two issues. These limitations may explain the same effect of the two resin cements on PBS in the present study. In the present study, the effects of E&R and SE cements on the PBS were not compared with that of the SA cement and should be evaluated in future studies.

Three types of nanoparticle solutions (SNP, ZNP, and TNP) were used as intraradicular dentin pretreatments to benefit from their antibacterial properties. In the present study, the effects of intraradicular dentin pretreatment with different nanoparticles on the PBS of fiber post to root dentin were evaluated. Silver is an antiviral and an important broad-spectrum antibacterial agent. It has been proposed that sustained silver ion release is responsible for the long-term antibacterial properties of silver. Moreover, silver has low toxicity and good biocompatibility with human cells especially in lower concentrations (12). The ability of SNP solution to prevent biofilm formation and reduce bacterial adhesion has already been confirmed. Therefore, it has been suggested as an antimicrobial irrigating solution in the preparation of the root canal for endodontic treatment (14). A previous study demonstrated that silver nanoparticles (25 nm) have a much higher antibacterial activity compared with zinc oxide (125 nm) and gold (80 nm) nanoparticles (23). Another nanoparticle which was applied as intarradicular dentin pretreatment in this study was ZNP. An inhibitory effect on the activity of matrix metalloproteinases (MMPs) which play a role in the degradation of dentin collagen has been demonstrated for zinc oxide (24). The stimulation of a metabolic effect in hard tissue mineralization, inhibition of dentin demineralization, and decreased decalcification resulting from orthodontic treatment have been reported following zinc oxide application (25,26). The effect of zinc oxide on collagen degradation in demineralized human dentin was much longer than that of chlorhexidine in a previous study (27). ZNPS have an improved antibacterial activity compared to zinc oxide due to the increased surface-to-volume ratio of the nanoparticles (28). Also, no adverse effect on bond strength to dentin was found for ZNPs (19).

TNPs were also applied as an intraradicular dentin pretreatment in the current study. In addition to their antibacterial effect by the production of free radicals, good antiadhesive properties against *Streptococcus mutans* were also demonstrated for TNPs in a previous research (29).

Compared with the control group, the PBSs of the E&R and SE resin cements used in this study significantly increased after intraradicular dentin pretreatment of the post space with SNP. This finding is in line with a previous study which showed that the application of SNP before fiber post cementation using an etch-and-rinse resin cement had the highest bond strength values and the lowest interface permeability in all thirds compared with the application of other irrigation solutions including distilled water, 25% polyacrylic acid, 2% chlorhexidine, and 5.25% NaOCl. This result was confirmed by scanning electron microscopy which demonstrated that the bonding process following irrigation with SNP might occur in the same way as that of the control group (6). No adverse effect on bond strength was found for dentin pretreatment with SNP, TNP, and ZNP in a previous study. Also, a higher coronal dentin bond strength value for SNP pretreatment was demonstrated compared to ZNP and TNP dentin pretreatments (19). The higher PBS of fiber posts following the application of SNP can be attributed to the water-based characteristic of SNPs. SNPs may provide an increase in the surface tension of the dentin substrate which might improve the penetration of the adhesive system through the etched intraradicular dentin when applied with E&R resin cement. Moreover, intraradicular dentin pretreatment might enhance the dentin wetness prior to the application of SE resin cement. Interestingly, intraradicular dentin pretreatment with SNP resulted in higher PBS values compared to the TNP-pretreated and ZNP-pretreated groups. The reason for this finding is not clear yet. The different interactions of the nanoparticles with root dentin might be related to the differences in the surface-to-volume ratio, morphology, chemical and colloidal stability, and aggregation stability of the NPs. Additionally, none of the nanoparticles applied as intraradicular dentin pretreatment interfere with the immediate PBS values. However, they might influence the long-term bond strength values which should be investigated in future studies. Also, the effect of intraradicular dentin pretreatment with different nanoparticles on the PBS of fiber posts was not dependent upon the type of the adhesive material used in this study.

In the present study, lower PBS values in the apical third were obtained compared to the middle or cervical third. Limited light access to this region and the resultant defective polymerization of the material could possibly justify this finding. Despite the higher ability of translucent glass fiber posts to transmit light than metal posts, the degree of conversion of the cement in the apical region was not comparable to those of the other regions (30). This finding was in line with a previous study (30). Other possible influencing factors on deficient sealing of the resin cement-dentin interface in the apical third are the presence of residual gutta-percha, incomplete dentin hybridization, and difficult moisture control in the apical region (30). In contrast to the present study, a previous study found no significant difference in the immediate PBS values of different root regions for an E&R resin cement and a SE resin cement (22).

Based on the results of the present study, using nanoparticle solutions especially SNPs before fiber post cementation would be beneficial in clinical practice because of their antibacterial properties. Moreover, they would not interfere with the adhesion of the fiber post. The present study has some limitations. No aging conditions (mechanical aging or thermal cycling) were carried out on the specimens. The effects of temperature changes, complex forces, and low intermittent functional forces from different directions in the oral environment should also be evaluated in future studies. Additionally, one etch-and-rinse cement and one self-etch resin cement were assessed in the present study, therefore, the obtained results cannot be generalized to all systems. Also, the probable release of nanoparticles into the oral cavity and saliva, the antibacterial properties, and long-term bond strength properties of the nanoparticles using various resin cements should be investigated in the future.

## Conclusions

According to the results of the present study, intraradicular dentin pretreatment with SNPs, TNPs, and ZNPs did not interfere with the PBSs of the fiber posts. The best result was obtained for the SNP-pretreated group compared to the control, TNP-pretreated, and ZNP-pretreated groups. The effect of SNP pretreatment on the improvement of the PBS of fiber posts was not material-dependent. Also, the lowest PBS was found in the apical third of the root canal spaces.
